# Household Income, Food Insecurity and Nutritional Status of Migrant Workers in Klang Valley, Malaysia

**DOI:** 10.5334/aogh.2859

**Published:** 2020-08-03

**Authors:** Chan Foong Mei, Erwin Martinez Faller, Lau Xiao Chuan, Jacklyn San Gabriel

**Affiliations:** 1 Faculty of Health and Life Sciences, Management and Science University, Shah Alam, MY; 2 Pharmacy Department, San Pedro College, Davao City, PH; 3 Faculty of Health and Social Sciences, Bournemouth University, UK; 4 College of Pharmacy, University of Southern Philippines Foundation, Cebu City, PH

## Abstract

**Background and Purpose:**

Food insecurity exists whenever accessibility to nutritious food is limited. It affects a person’s health with regards to nutritional status, indicated by malnourishment or overnutrition. This study aims to study the relationship between household income, household food insecurity, and weight status of migrant workers in Klang Valley, Selangor.

**Method:**

A cross-sectional study involving a convenience sampling of 125 documented migrant workers from five selected countries was conducted. A researcher-administered questionnaire consisting of socio-demographic questions, three-day 24-hour dietary recall (3DR), and nine-item Household Food Insecurity Access Scale was used. Anthropometric measurements, including body weight, height, and waist circumference, were taken.

**Findings:**

About 57.6% of the households studied were food insecure (24.8% mildly, 29.6% moderately, and 3.2% severely). Burmese were found to have the highest rate of household food insecurity (96%). The majority of the migrant workers were of normal weight (68.0%). No significant relationship was found between monthly household income and household food security status (*p* = 0.475), as well as between household food security status and weight status (*p* = 0.535).

**Conclusion:**

Results imply that food security status affects certain nutrient intake among migrant workers. There were no significant associations between variables. Interventions focusing on nutritional education on food choices and implementation on health policy are recommended. Further studies should consider the accessibility, nutritional-related diseases, and dietary aspects of migrant workers, which are risk factors for food insecurity.

## Introduction

Food insecurity is a significant nutritional issue that occurs worldwide and commonly found in low-cost households in many developed and developing countries, especially in the Asian region [1]. According to Life Sciences Research Office (1990), food insecurity arises whenever the availability or accessibility to nutritious and safe food is limited, and when there is an inability to acquire socially and culturally acceptable food in order to live a healthy and active life. At the household level, the concept also consists of the availability, accessibility, sufficiency, sustainability, and security of food supply [2]. It refers to the ability of a household to secure sufficient food to meet the dietary requirements of every household member, either by means of its own food production or purchase [3].

Food insecurity may have adverse impacts on the nutritional and health status of individuals, and these impacts may vary between high income and low to middle-income countries [4]. At the household level, there is always an association between food insecurity and low income, poverty, insufficient and imbalance food intake as well as poor weight status. Although studies on the relationship between household food insecurity and nutritional status of Malaysians are well-established [1,2], there is no prevalent study on these variables among Migrant workers in Malaysia. Hence, this study is designed to investigate the relationship between these factors among Migrant workers, which may serve as a basis for an appropriate intervention plan.

## Methodology

### Research Design

A quantitative approach, nonexperimental, cross-sectional study design was adapted to describe the household income, food insecurity, and nutritional status of migrant workers in Malaysia. Three researchers collected, analysed, and tabulated the data obtained using demographic profile sheet, three-day 24-hour dietary recall (3DR), Household Food Insecurity Access Scale (HFIAS), and anthropometric measurements.

Ethical approval was granted by the Ethical Committee from Management and Science University (MSU-RMC-02/FR01/08/l1/0610). Written informed consent was obtained from the respondents prior to data collection. Respondents were administered by the researcher to answer the questionnaire as anonymous. Only those who were interested and willing to participate in this study were recruited based on the ethical considerations of the research. Self-selected study participants have been found to give more accurate, and frank answers [5].

### Research Locale, Population, and Sampling

The research was done in five location sites such as non-government organizations offices, churches, and workplaces; wherein migrant workers can be found within Klang Valley, Selangor. The researcher decided to randomly select 25 documented migrant workers from Indonesia, Bangladesh, Myanmar, Nepal, and the Philippines. A total of 125 documented workers aged 19 to 55 years were selected. Subjects with physical or mental complaints were excluded from this study, as they could influence the results of the validation. Out of 150 available subjects, only 125 subjects passed the inclusion and exclusion criteria.

### Data Collection

#### Socio-Demographic and Socio-Economic Background

At baseline, a researcher-administered socio-demographic and socio-economic questionnaire was used to collect information on age, gender, marital status, occupation, educational level, household size, personal monthly income, monthly household income, and income per capita.

#### Three-Day 24-hour Dietary Recall (3DR)

The 3DR method was selected as it was more convenient and time-efficient for the subjects. It was administered individually for each meal per day for three days, which consisted of two days during the weekdays and one day on the weekend. In the 3DR, subjects were asked about the food they ate, portion size, preparation method, and the ingredients used.

#### Household Food Insecurity Access Scale (HFIAS)

A 9-item HFIAS was used to measure the food security status of the subjects adapted from Food and Nutrition Technical Assistance (FANTA) [6]. HFIAS Score and HFIAS Prevalence were used to report the household food insecurity degree in the past four weeks (30 days). The maximum score for a household is 27, while the minimum score is 0. The higher the score, the more food insecurity (i.e., access) the household experienced. In contrast, the lower the score, the less food insecurity (i.e., access) the household experienced. The HFIAS prevalence, categorizes the households into four levels of household food insecurity (access), namely food secure, and mildly, moderately and severely food insecure. Households were categorized as increasingly food insecure if they responded affirmatively to more severe conditions and/or experienced those conditions more frequently.

#### Anthropometric Measurements

Anthropometric measurements were taken to obtain the body mass index (BMI) of each subject. A portable TANITA weighing scale was used to measure the subjects’ weight to the nearest 0.1 kg, while a SECA Body Metre was used to measure the subjects’ height to the nearest 0.1 cm. BMI was calculated using the special formula, BMI = weight (kg)/height^2^ (m^2^). Waist circumference was measured with a flexible but non-stretchable measuring tape to the nearest 0.1 cm.

#### Statistical Analysis

The collected data was analyzed using the Statistical Package for Social Science, version 20.0 (IBM, New York, USA). Dietary intake was analyzed using Nutritionist Pro™ Diet Analysis Software version 3.1.0. Descriptive analyses of the data were used to obtain means and standard deviations (SD) for continuous variables, as well as frequencies and percentages for categorical variables. Kruskal-Wallis test and Mann-Whitney U test were used for non-parametric data, whereas Crosstab Chi-square was used to examine the relationship between categorical variables. Pearson R regression analysis was also used to identify the association between food security towards HMI and BMI or nutritional status. A statistical probability level of p < 0.05 (two-sided) is considered significant.

## Results

### Socio-demographic and socio-economic background

The mean age of the subjects was 32.49 ± 7.85 years (Table 1). 60% males and 40% of females were involved, with each nationality making up 20% of the subjects in this study. About 60% of the subjects had been working for more than three years in Malaysia. The average household size of the subjects was 5.63 ± 1.43, and the mean monthly household income was RM 1784.61 ± 575.48. Of the households, 14.4% belong to the very low-income group, earning no more than RM 1000 per month. The average income per capita of the subjects was RM 327.48 ± 110.44. With regard to the priority of salary spending, 48.8% of the subjects chose to send the money back to their hometown upon receiving their salary before spending it on other expenses such as house rental and utility bills.

**Table 1:** Demographic and socio-economic characteristic of subjects (*n = 125*).

Variable	n	Percentage (%)	Mean ± SD

Age (years)			32.49 ± 7.85
Gender			
Male	75	60.0	
Female	50	40.0	
Marital status			
Single	44	35.2	
Married	75	60.0	
Divorced	3	2.4	
Widowed	3	2.4	
Religion			
Islam	50	40.0	
Buddhist	27	21.6	
Hindu	22	17.6	
Christian	26	20.8	
Others	0	0	
Nationality			
Indonesia	25	20.0	
Bangladesh	25	20.0	
Myanmar	25	20.0	
Nepal	25	20.0	
Philippines	25	20.0	
Living area			
Village	97	77.6	
City	28	22.4	
Occupation			
Construction worker	29	23.2	
Cleaning worker	9	7.2	
Domestic worker	20	16.0	
Security guard	15	12.0	
Factory worker	29	23.2	
Foodservice worker	15	12.0	
Others	8	6.4	
Working experience in Malaysia			
0 month–1 year	25	20.0	
2 years–3 years	25	20.0	
4 years–5 years	33	26.4	
6 years–7 years	20	16.0	
>7 years	22	17.6	
Household size			5.63 ± 1.43
1–5	64	51.2	
6–10	60	48.0	
≥11	1	0.8	
Number of children			1.32 ± 1.32
Personal monthly income (RM)			1215.12 ± 260.23
Household monthly income (RM)			1784.61 ± 575.48
≤1000	18	14.4	
1001–1600	32	25.6	
1601–2200	51	40.8	
2201–2800	19	15.2	
2801–3400	5	4.0	
Income per capita (RM)			327.48 ± 110.44
First priority on spending salary			
Sending back to hometown	61	48.8	
House rental	52	41.6	
Electricity and water	12	9.6	

### Household Food Insecurity

**Figure 1:**
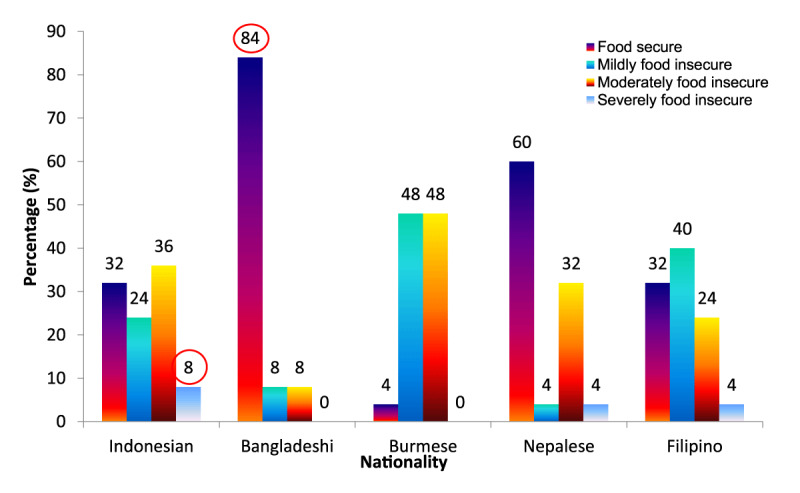
Household food security status of migrant workers by nationality (n = 125).

Figure 1 shows the food security status of migrant workers by nationality. 84.0% of Bangladeshi and 60.0% of Nepalese workers were found to be food secure, while 8.0% of Indonesian workers, as well as 4.0% of Nepalese and Filipino workers, were severely food insecure; 48% of Burmese workers were both mildly and moderately food insecure.

### Weight Status

**Figure 2:**
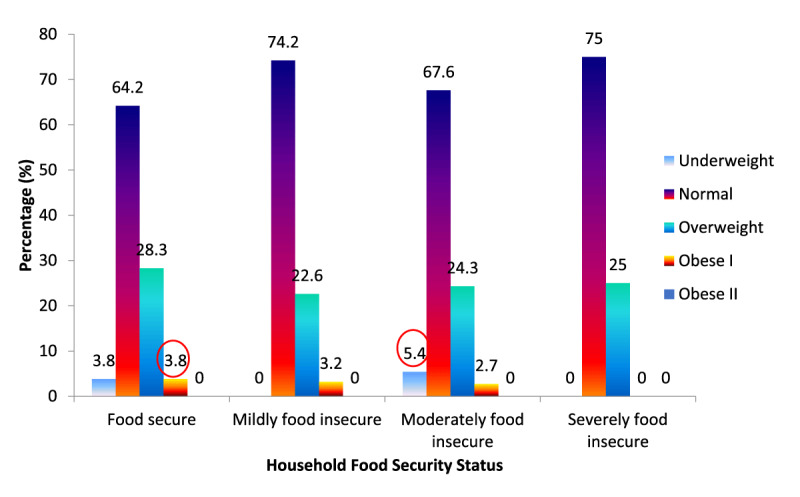
Weight status of migrant workers by nationality (n = 125).

Figure 2 shows that Burmese workers comprised the majority of subjects with a normal BMI (80%) compared to other nationalities. No underweight case was found among Nepalese workers, but for each of the other nationalities, 4% of the subjects were underweight. The largest group of overweight workers was the Bangladeshi group of workers (40%). There was an obese class I cases among three nationalities: Indonesians (12%), Bangladeshis (4%), and Filipinos (4%). None was categorized under the obese II category (0%).

### Nutrient Intake

The mean calorie intake among the subjects was *1952.34* ± 662.90 kcal (Table 2). For the macronutrients, the mean values for carbohydrate, protein, and total fat were *310.11* ± 122.86 g, *72.29* ± 24.66 g, and *47.44* ± 21.50 g, respectively. The mean of Vitamin C intake among the subjects was *32.80* ± 25.93 mg. With regard to mineral intake, calcium and iron showed a mean of *362.87* ± 250.69 mg and *14.04* ± 6.19 mg, respectively. Meanwhile, the mean value for dietary fibre intake for the subjects was *2.36* ± 1.45 g.

**Table 2:** Nutrient intakes of migrant workers (*n = 125*).

Variable	Mean ± *SD*

Energy (kcal)	1952.34 ± *662.90*
Carbohydrate (g)	310.11 ± *122.86*
Protein (g)	72.29 ± *24.66*
Fat, total (g)	47.44 ± *21.50*
Vitamin C (mg)	32.80 ± *25.93*
Calcium (mg)	362.87 ± *250.69*
Iron (mg)	14.04 ± *6.19*
Dietary fiber, total (g)	2.36 ± *1.45*

### Relationship between Household Monthly Income and Household Food Security Status

Chi-square test and Pearson correlation showed no significant relationship between monthly household income and household food security status at p = 0.540 and r = –0.247, respectively (Tables 3 and 5). Among the four groups, the highest percentage with ≤RM1000 monthly household income was found in the severe food insecure group (20.8%), while the number of workers with a monthly household income of RM2801–RM3400 was the highest in the moderate food insecure group (5.4%).

**Table 3:** Household monthly income of migrant workers by household food security status (*n = 125*).

Variable	Food Secure (n = 53)	Mildly Food Insecure (n = 31)	Moderately Food Insecure (n = 37)	Severely Food Insecure (n = 4)	X^2^ value	p-value

Household monthly income (RM)					5.030	0.540
≤1000	11 (20.8)	2 (6.5)	4 (10.8)	1 (25.0)		
1001–1600	17 (32.1)	8 (25.8)	7 (18.9)	NA		
1601–2200	18 (34.0)	13 (41.9)	17 (45.9)	3 (75.0)		
2201–2800	5 (9.4)	7 (22.6)	7 (18.9)	NA		
2801–3400	2 (3.8)	1 (3.2)	2 (5.4)	NA		

NA = Not Applicable. Chi-square test, p < 0.05.

### Relationship between Weight Status and Household Food Security Status

Chi-square test and pearson correlation showed no significant relationship between weight status and household food security status at p = 0.535 and r = 0.109, respectively (Tables 4 and 5). In the severe food insecure group, there were a majority of normal weight (75.0%) and less overweight (25.0%) cases. There were no underweight cases in the mild food insecure group and severely food insecure group (0%).

**Table 4:** Weight status of migrant workers by food security status (*n = 125*).

Variable	Food Secure (n = 53)	Mildly Food Insecure (n = 31)	Moderately Food Insecure (n = 37)	Severely Food Insecure (n = 4)	X^2^ value	p-value

BMI (kg/m^2^)					7.995	0.535
Underweight	2 (3.8)	NA	2 (5.4)	NA		
Normal	34 (64.2)	23 (74.2)	25 (67.6)	3 (75.0)		
Overweight	15 (28.3)	7 (22.6)	9 (24.3)	1 (25.0)		
Obese I	2 (3.8)	1 (3.2)	1 (2.7)	NA		

BMI: Body Mass Index. NA = Not Applicable. Chi-square test, p < 0.05.

**Table 5:** Association of Food Insecurity, Household Income, and Nutritional Status among Migrant Workers.

Test Variables	Pearson R correlation	Description	P value	Remarks

Food Insecurity & Household Monthly Income	–0.247	Very low correlation	0.294	Not significant
Food Insecurity & Nutritional Status	0.109	Very low correlation	0.687	Not significant

## Discussion

Based on this study, households with lower income were not at risk for household food insecurity. This finding supports the study conducted by Nord and Brent (2002), as food insecurity exists among middle- and high-income households [7]. Wirth et al. 2007 found that income was the single greatest predictor of hunger and food insecurity, followed by migratory status but in the case of this study since it has been established that there is no significant relationship between monthly household income and household food security status, it compels the researchers to look at other probable risk factors that contribute to the food insecurity status of these respondents [8]. This may be due to the misunderstanding of questions, random or erratic responses to the survey, or the existence of high expenditures in a household. On the other hand, there might be unusually high economic needs in the household among the subjects. Some households have either episodic or chronic needs that deplete economic resources [7].

According to the Institute of Medicine and National Research Council (2013), those who live at or below the poverty threshold, access to healthy foods at a reasonable price is a challenge that often places a strain on already limited resources and may compel them to make food choices that are contrary to current nutritional guidelines [9].

Since 48.8% of the subjects chose to send the money back to their hometown upon receiving their salary before spending it on other expenses such as house rental and utility bills, it drives the respondents to a stringent way of living. They would opt for buying groceries, which is cheaper than eating on fast-food restaurants and has higher probabilities of eating home-cooked meals with more nutritional value [6,10,11]. Borre et al. (2010) cited that families who spent a larger percentage of their income on food were food secure compared to those who were food insecure, both with and without hunger, for both adults and pre-school aged children [12]. The other half of the respondents may have prioritized on food more than other obligations which could be a probable reason for their food security status despite a low income [13,14,15,16,17]. Other factors for their food security on low-income respondents that is beyond the scope of this study could be that they are enrolled in certain nutrition assistance programs available in Malaysia which is designed to improve access to healthy foods for low-income individuals and households. Thus, increasing the food purchasing power of low-income families [18].

In contrast, this finding is different than that of other studies. Previous studies commonly found that households with lower income are at risk for food insecurity. This was also supported by the study conducted by Zalilah and Tham (2002), which found that when the household income and income per capita increased, household food security subsequently improved [19]. The study by Ihab et al. (2013) revealed a negative association between total household income and household food insecurity status, showing that an RM10 rise in the monthly household income will reduce the odds of being food insecure by 3% [20]. Likewise, the more money a household has, the greater the access is to better food in terms of quality or quantity. The relationship between income and household food security is a sequential relationship between food expenditure and diet diversity, which leads to food security [21]. Among the poor households, insufficient income can contribute to the inability to provide adequate food for members of the household [1].

In this study, the anthropometric measurements indicated that many subjects were of normal weight and at low risk for abdominal obesity. More females were at risk for abdominal obesity than males [22,23]. Several studies in Malaysia on the Malaysian population have also reported a higher prevalence of overweight, obesity, and the risk for abdominal obesity among females compared to males. A possible reason for this prevalence was the association with lower quality of diet (higher intake of carbohydrates and a lower intake of fruits and vegetables), and low to moderate physical activity may also contribute to the accumulation of body fat, consequently leading to the risk of overweight, obesity and abdominal obesity [1]. Mostly male (60%) in the study generally works as construction and factory workers that needed higher physical energy, which lessens the possibility of overweight and obesity in comparison with the security guard and food service line.

In terms of food security status, this study found that food insecurity did not have an impact on BMI, or no relationship could be established between BMI and household food security status. This point towards the existence of additional risk factors, some of which have been elucidated and validated by empirical literature. Based on the study of Borre et al. (2010), all participants reported being concerned about overweight and the development of obesity among their children. Overweight and obesity were prevalent among all food security groups of adults, and those children who were food insecure were less overweight and obese than those who were food secure [12]. The participants related their obesity problem to their lifestyle as farmworkers because they had no alternative explanation. They attributed their obesity to family history, lack of exercise and eating fried foods or sweets, categories of individual qualities and behaviors [8,24].

In contrast, according to Townsend et al. (2001), food insecurity was related to overweight status for women (n = 4509, p < 0.0001), but not for men (n = 4970, p = 0.44) [25]. Besides that, food insecurity with hunger was associated with an increased risk for obesity among Asians, Blacks, and Hispanics (OR = 2.81) but not for non-Hispanic Whites (OR = 0.82) [26]. In a study conducted by Thuy et al. (2015) in Vietnam, there existed a trend where a higher fraction of subjects from food-insecure households were overweight or obese compared to their food-secure counterparts [4]. This finding supports previous evidence which suggested that food insecurity and obesity coexist in disadvantaged households in the urban areas of developing countries [27]. This might be due to the continuous economic integration of the developing countries, with the international trading market, causing the cost of processed food with lower nutritional quality to become less expensive. Cheaper prices may lead to increased consumption of such food, which, when accompanied by lower levels of physical activity, may contribute to food insecurity, and consequently, the obesity paradox in urbanized areas.

In terms of dietary intake, the data showed that migrant workers had low intakes of energy, and most of the nutrients were below the recommended levels. The reasons for dietary change with immigration were related primarily to food access, availability, and cost as well as the organization of work [12,28,29]. Obtaining food was always dependent on having a regular income, transportation to the store and a time and place to prepare food [30,31,32,33]. Some food, like cheese, dairy, and fresh vegetables were considered too expensive to buy in supermarkets, but there may be no local, inexpensive sources of this food near their vicinity to some of the respondents.

Dietary changes commonly mentioned were increasing the amount and frequency of meats, drinking sodas, eating processed foods, and snacks [13,34,35]. Other probable factors to look into could be meal patterns were affected by their work schedule, which left little time to prepare foods or eat meals. The migrant workers may return home from work feeling tired and without much time to prepare a meal, especially in a household where young children required attention. Working conditions such as being at work for long hours could reduce access to fresh foods and poor living conditions; thus, they were not able to prepare nutritious and safe meals on a daily basis. Also, due to working conditions, it could lead to workers bringing leftovers or packaged foods from home to work and ate hurriedly unless the work was slow, and they could take a break. If work was competitive or very busy, they did not break except to drink some water or use the bathroom. Time and money often determined what they ate and can be seen as a trade-off dilemma [12,17,27,34,35].

Household food insecurity was associated with the consumption of poor quality diets among adults [36]. The mean for protein and fat intakes decreased significantly as food insecurity worsened (p < 0.05). Several studies have reported that food-insecure households have lower nutrient intakes, diet diversity or variety, and the number of servings from the expensive food groups (meat/fish/poultry/legumes as well as fruits and vegetables) compared to food-secure households due to limited household income [37,38]. In addition, there were studies that reported that lower intakes of Vitamin C, calcium, and iron were found among the food insecure than food-secure subjects [39]. Findings from all of these studies indicate that the food insecurity experience is likely to affect the quality and quantity of diet as well as the eating behaviour of the subjects.

Although there were no significant differences between energy and carbohydrate intake and food security status, there was an observed trend where the energy and carbohydrate intake decreased as food insecurity worsened. This finding is supported by several studies which have shown that the diet of the subjects reporting household food insecurity or food insufficiency was not only inadequate in nutrient and food variety, but also in calorie [39,40,41]. Food insecurity is a broader term that encompasses food insufficiency, as well as psychological and qualitative aspects of food supply and intake. Living in a food-insecure household carries serious health implications. Borre et al. 2010 reported in their study that research among adults with food insecurity has demonstrated associations between food insecurity and poor physical and mental health, poorer self-reported health status, and diabetes mellitus [12]. Kiehne and Mendoza, (2015) stated based on their findings that migrant and season farmworkers demonstrated that food insecurity has a negative impact on overall health and is correlated with a number of conditions, including birth defects, diabetes, chronic diseases, and depression [42]. Food insecurity is also linked with a greater likelihood of being overweight, obese or diabetic, perhaps due to consumption of less nutritious food and more junk food; this, in turn, deteriorates physical health, emotional health, and overall well-being further [24,34,36,43,44,45].

## Limitations

The small number of subjects might have caused a lack of accuracy in the results. The communication barrier was also another factor that existed between the researcher and subjects during data collection. This might have caused a misunderstanding of the questions when answering the questionnaire, which could possibly have produced inaccurate results. The date of this study is cross-sectional; therefore, they do not allow causal judgment between food security and other risk factors such as household income and nutritional outcomes. Small sample size prevents results from being generalized to all migrant workers in Klang Valley, Malaysia; however, food security findings are similar to other studies with larger samples [1,12,36].

Relationships among food security and obesity, food security and migration, and food security and income status of the sample were not able to be determined from this study.

## Conclusion

The majority of the migrant workers are food secure, with normal weight status. Some of the migrant workers are at risk for abdominal obesity, and females are at higher risk than males. Their dietary intake is affected by household food security status. No relationship between household income, household food security status, and weight status was found among the migrant workers in this study. Interventions focusing on nutritional education on food choices and implementation on health policy are recommended. Further studies should consider the accessibility, nutritional-related diseases, and dietary aspects of migrant workers, which are risk factors for food insecurity.
